# Gradient boosting machine learning to improve satellite-derived column water vapor measurement error

**DOI:** 10.5194/amt-13-4669-2020

**Published:** 2020-09-02

**Authors:** Allan C. Just, Yang Liu, Meytar Sorek-Hamer, Johnathan Rush, Michael Dorman, Robert Chatfield, Yujie Wang, Alexei Lyapustin, Itai Kloog

**Affiliations:** 1 Department of Environmental Medicine and Public Health, Icahn School of Medicine at Mount Sinai, New York, New York, USA; 2 Universities Space Research Association (USRA), Mountain View, California, USA; 3 NASA Ames Research Center, Mountain View, California, USA; 4 Department of Geography and Environmental Development, Ben-Gurion University of the Negev, Beersheba, Israel; 5 Joint Center for Earth Systems Technology, University of Maryland, Baltimore County, Baltimore, Maryland, USA; 6 NASA Goddard Space Flight Center, Greenbelt, Maryland, USA

## Abstract

The atmospheric products of the Multi-Angle Implementation of Atmospheric Correction (MAIAC) algorithm include column water vapor (CWV) at a 1 km resolution, derived from daily overpasses of NASA’s Moderate Resolution Imaging Spectroradiometer (MODIS) instruments aboard the Aqua and Terra satellites. We have recently shown that machine learning using extreme gradient boosting (XGBoost) can improve the estimation of MAIAC aerosol optical depth (AOD). Although MAIAC CWV is generally well validated (Pearson’s *R* >0.97 versus CWV from AERONET sun photometers), it has not yet been assessed whether machine-learning approaches can further improve CWV. Using a novel spatiotemporal cross-validation approach to avoid overfitting, our XGBoost model, with nine features derived from land use terms, date, and ancillary variables from the MAIAC retrieval, quantifies and can correct a substantial portion of measurement error relative to collocated measurements at AERONET sites (26.9% and 16.5% decrease in root mean square error (RMSE) for Terra and Aqua datasets, respectively) in the Northeastern USA, 2000–2015. We use machine-learning interpretation tools to illustrate complex patterns of measurement error and describe a positive bias in MAIAC Terra CWV worsening in recent summertime conditions. We validate our predictive model on MAIAC CWV estimates at independent stations from the SuomiNet GPS network where our corrections decrease the RMSE by 19.7% and 9.5% for Terra and Aqua MAIAC CWV. Empirically correcting for measurement error with machine-learning algorithms is a postprocessing opportunity to improve satellite-derived CWV data for Earth science and remote sensing applications.

## 1 Introduction

Water vapor represents a small but environmentally significant constituent of the atmosphere. The integrated water vapor from ground to space is defined as the column water vapor (CWV), in units of centimeters (i.e. precipitable water vapor; Gao and Goetz, 1990). CWV has important applications in many fields, such as atmospheric correction of remote sensing images, Earth energy balance and global climate change, land surface temperature retrieval in thermal remote sensing, and astronomy. Thus, high resolution CWV values with global coverage have multiple uses in Earth science and remote sensing. CWV has been measured by multiple technologies and monitoring networks, including sun photometers, GPS sensors (e.g. SuomiNet), Aerosol Robotic Network (AERONET) sun photometers, and satellite remote sensing. The AERONET sun photometer network measures CWV at approximately 400 stations worldwide, in channels centered at 940 nm and provided to the user in Level 2, which is the highest data-quality level provided by AERONET (Pérez-Ramírez et al., 2014). The AERONET CWV data have been well validated with the U.S. Department of Energy Atmospheric Radiation Measurement (ARM) program radiosonde observations and other ground-based retrieval techniques, such as microwave radiometry (MWR) and SuomiNet GPS receivers, and do not observe any dependence of biases with the zenith angle (Pérez-Ramírez et al., 2014). AERONET CWV has been used in studies that examine aerosol optical, microphysical, and radiative properties in Africa (Adesina et al., 2014; Boiyo et al., 2019; Kumar et al., 2013), the Brazilian tropics (Schafer et al., 2008), and Beijing and Kanpur (Wang et al., 2011). Global satellite-borne CWV is available at a high resolution (1 km), from the Multi-Angle Implementation of Atmospheric Correction (MAIAC) algorithm derived from daily overpasses of NASA’s Moderate Resolution Imaging Spectroradiometer (MODIS) instruments aboard the Aqua and Terra satellites. The MAIAC CWV is computed using MODIS near-infrared (NIR) channels centered at 940 nm. This method applies two ratios of channels to compute the water vapor transmittance and then compute the amount of water vapor using lookup tables (Lyapustin et al., 2014). The MAIAC CWV algorithm was validated against ground measurements of CWV from 265 AERONET stations worldwide, with a relatively strong association (Pearson’s *R* >0.95; root mean squared error (RMSE) < 0.25 cm; average accuracy of ±15%; Martins et al., 2019). These datasets were collocated by averaging MAIAC values within 9 pixels × 9 pixels and AERONET values ±30 min of the satellite overpass in cloud-free conditions. A significant upward trend (*p* <0.05) for MAIAC TERRA was found over most regions, although this was not significant over Northeastern USA. Globally, the highest average correlation between MAIAC CWV retrievals from both Aqua and Terra with AERONET CWV has been shown in Asia and both the Northern and Southern regions of the USA.

In spite of the strong performance of MAIAC CWV in multiple locations, comparing it with collocated AERONET CWV, there may be opportunities to characterize and correct complex interactions and challenging conditions that increase satellite retrieval error. However, it has not yet been assessed whether machine-learning approaches can improve the estimation of satellite-borne CWV. We have recently demonstrated that machine learning using extreme gradient boosting (XGBoost; Chen and Guestrin, 2016) can improve the estimation of MAIAC aerosol optical depth (AOD) parameters over AERONET stations (43% decrease in cross-validated RMSE; Just et al., 2018). For an introduction to gradient-boosted regression trees, please see the work of Elith et al. (2008). XGBoost involves fitting a large number of tree-based models. Each subsequent tree is fit to the error from the previous trees, and the predictions of all the trees are added together. Each tree’s prediction is multiplied by a shrinkage factor (or “learning rate”) *η*, a number between 0 and 1. By adding successive trees, XGBoost descends the gradient of the loss function. The component trees use a recursive binary partitioning of the predictors that accommodates varying types and scales of predictor variables and is robust to outliers (Elith et al., 2008). An advantage of flexible algorithmic machine-learning approaches such as XGBoost is that they can model complex phenomena (Chen and He, 2015), including interactions of multiple features (e.g., retrieval angles, seasonality, and surface characteristics). The resulting prediction model can be used as an algorithm to reduce the retrieval error. Machine-learning tools for model interpretation can also help explain the contributions of these features to retrieval error and guide feature selection to build parsimonious models.

While the satellite data record continues to grow, the ground monitoring networks that can be used for validation and algorithmic measurement error correction of satellite retrieval products are still sparse. Collocated ground-satellite datasets may thus have important nonindependent spatiotemporal structures if they rely on observations that occur in only a few locations. Flexible machine-learning models would overfit to the characteristics of these particular stations or the days when AERONET data are available if cross validation assumed independence of observations. While machine-learning applications in aerosol research have begun to adopt group K-fold cross validation for assessing model fit across fixed monitoring networks (Di et al., 2016), we propose a novel cross-validation approach taking into consideration data structure due to both fixed sites and the correlation of observations from the same day.

The goals of this work are to (1) evaluate whether machine-learning gradient boosting models can improve satellite-based CWV retrievals and (2) understand the contributions of different features as well as spatial and temporal structures of the ground station measurements to predict error in the estimated CWV. The data and machine-learning methods are described in Sect. 2, followed by a discussion of the results in Sects. 3 and 4.

## 2 Description of the data

**Figure 1.**
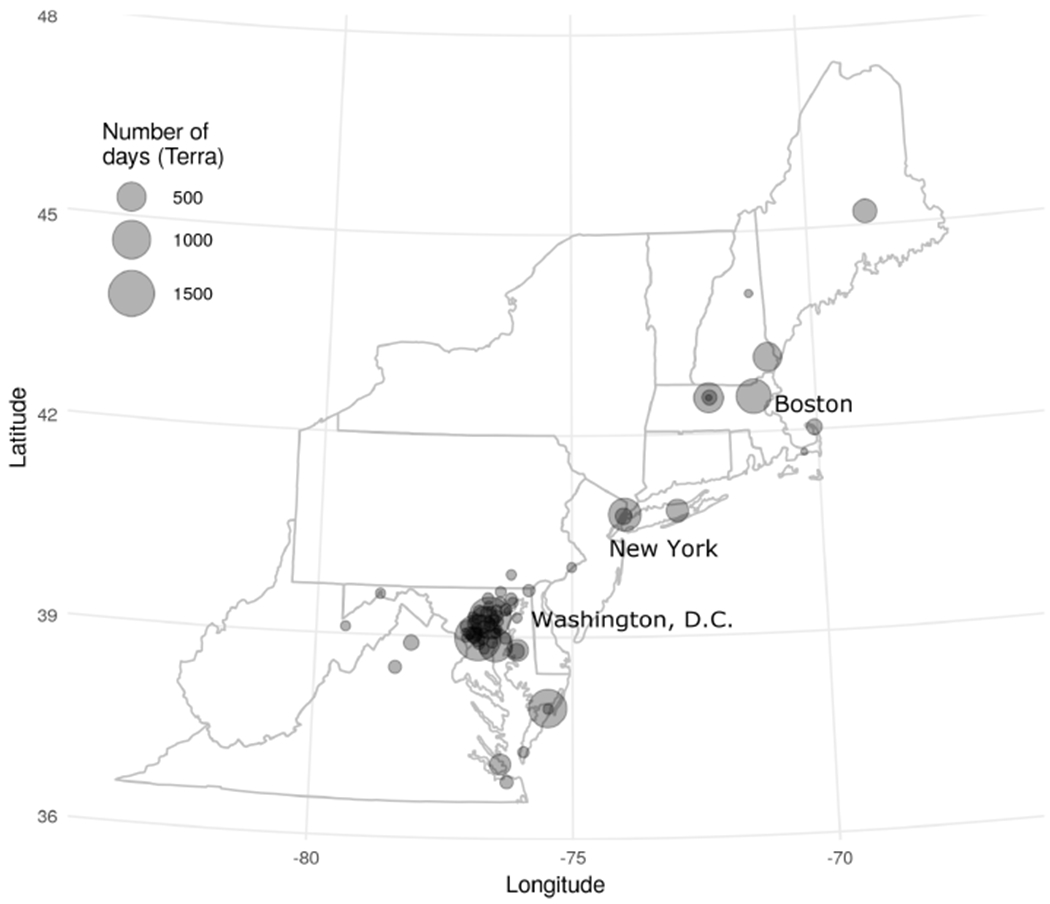
Study region in Northeastern and Mid-Atlantic USA with 75 unique AERONET stations showing the number of days with observations from the collocated Terra dataset.

In order to assess the agreement of the MAIAC estimates of CWV with those from AERONET, collocated datasets were built using MAIAC data (Lyapustin and Wang, 2016) where AOD was available (representing clear-sky conditions) from both Terra and Aqua (separately) collocated to the nearest 1 km × 1 km grid centroid and the closest observation in time (no more than 60 min) with cloud-screened (version 2, Level 2.0; Smirnov et al., 2000; Aerosol Robotic Network, 2019) measurements of CWV from the AERONET network of sun photometers over Northeastern USA (including 13 states and the District of Columbia from Maine to Virginia). The study period included 10247 observations (from 75 AERONET stations) for Terra (2000–2015) and 8536 observations (from 71 stations) for Aqua (2002–2015). All analyses were performed for Terra and Aqua datasets separately. AERONET stations in the northeast are largely urban and coastal (Fig. 1). We defined our target modeling parameter as the difference between MAIAC and AERONET CWV (ΔCWV = MAIAC CWV − AERONET CWV) such that any variation from zero indicated a component of measurement error that we sought to explain.

After exploratory scatterplots of ΔCWV versus time showed a temporal cluster of large outliers coming from a single AERONET station (City College of New York), observations from this site between 17 June 2007 and 1 January 2009 were dropped from further analysis, including 99 observations collocated for Terra and 95 observations for Aqua datasets. This particular period, which was flanked on both sides with months without values at that station, showed a clear deviation from the monitor’s typical trend across the remainder of the study period.

The date range for the collocated Terra dataset was from 25 February 2000 to 27 December 2015, including observations from 3024 unique days (52% of days during this interval). The date range for the collocated Aqua dataset was from 4 July 2002 to 28 December 2015, including observations from 2627 unique days (53% of days during this interval).

## 3 Statistical methods

We examined the use of XGBoost (Chen and Guestrin, 2016) for improving satellite-based MAIAC CWV retrievals and decreasing estimation error, as this method had previously outperformed two related supervised learning approaches using regression trees, namely gradient boosting and random forests, in a similar application (Just et al., 2018). The XGBoost algorithm is a popular implementation of boosted regression trees (Friedman, 2001). For an introduction to regression trees, see Strobl et al. (2009). A regression tree is a model that specifies recursive binary splits of predictors and assigns a constant value to all cases that end up in the same terminal node (namely, their mean on the dependent variable). The algorithm chooses the splits across all predictors that minimize the variance of the residuals. The maximum number of splits within each tree (also known as the maximum depth) can be set as a hyperparameter. A set of multiple trees can be used for prediction by combining the outputs of the individual trees for each case. Such a set of trees can accommodate complex relationships including nonlinearities and interactions while being robust to outliers. Boosting is a method of fitting a series of models iteratively, with each model fit on the residuals of the previous models. While each tree may individually perform relatively poorly at predicting the outcome (and thus is known as a “weak learner”), the combination of many trees can collectively describe complex relationships and account for the impact of many predictors. Further, because boosting includes sequentially learning by combining many iteratively fit trees that address the error in previous trees, this technique performs well, achieving low testing error. The XGBoost package is a scalable gradient boosting implementation with additional features including penalties to avoid overfitting and optimized computational speed (Chen and Guestrin, 2016).

We end up with more parsimonious XGBoost models, i.e., fewer trees, by adopting the concept of “dropout” from deep learning, in which individual learners are randomly dropped during training. Specifically, we used Dropouts meet Multiple Additive Regression Trees (DART; Rashmi and Gilad-Bachrach, 2015). Dropping trees helps to avoid the diminishing contributions and overspecialization of later trees in XGBoost. This is particularly important in our application given the low number of AERONET stations and relatively small size of the collocated datasets for machine-learning algorithms. XGBoost has several hyperparameters related to the desired size and complexity of the model that need to be set in training for each dataset. We had a priori selected to tune our XGBoost models with DART using six hyperparameters (Supplement Table S2), while using default values for other potential hyperparameters based on previous modeling experience. Our tuning and evaluation approach used two-level (nested) cross validation. Within each training fold for our outer cross validation, we further randomly split the training data in half and performed a 2-fold cross validation to compare the performance of XGBoost models using 50 random sets of potential hyperparameters selected with Latin hypercube sampling (Stein, 1987) to be well spaced across the range of potential hyperparameter values. While this is more similar to a random search than a grid search, it is expected to more efficiently find well-performing sets of hyperparameters than random search does, because it decreases the likelihood of checking combinations that are trivially different or leaving unexplored regions in the six-dimensional space, which has too many combinations to effectively cover with a grid search. We selected the set of hyperparameters that minimized the RMSE within the withheld portion of the training data before refitting with all training data.

Prior to feature selection, initial analyses included 25 candidate features such as MAIAC variables including an uncertainty parameter related to blue band surface reflectance, relative azimuth angle, and AOD; time trend (integer date); elevation (United States Geological Survey, 2018); several land use terms from the National Land Cover Database 2011 (Multi-Resolution Land Cover Consortium, 2017) aggregated to the proportions within 1 km × 1 km grid cells as well as the proportion of water within 5–15 km buffers; and distance to major water bodies (the Great Lakes and the Atlantic Ocean). Feature engineering calculated candidate features based on spatial patterns in nonmissing MAIAC data including the number of contiguous nonmissing grid cells (clump size) and the number of nonmissing observations in focal windows of side lengths from 30 to 510 km. Details on the data sources and feature engineering for all candidate features are included in the Supplement. No external meteorology or assimilated data were included.

The contributions of each feature to cross-validated predictions were estimated from Shapley Additive Explanations (SHAP) values (Lundberg et al., 2018). These SHAP values form an additive feature attribution measure to interpret complex machine-learning models. SHAP values estimate the contributions of each feature to each individual prediction (for ΔCWV, this is in units of centimeters). Specifically, the SHAP value for a given predictor and a given observation is the difference in the output, i.e., a predicted ΔCWV, depending on if the model is fit with or without the predictor. For each observation, the sum of all SHAP values, plus the bias term (the overall mean of ΔCWV), equals the prediction from the XGBoost model. The resulting matrix of SHAP values can be summarized to understand how a predictor contributes to the predictions. The mean absolute SHAP value across all observations summarizes the global feature importance, and more local model interpretation is possible through exploratory data visualizations such as scatterplots of individual predictors versus their SHAP values.

Because a more parsimonious set of features can ease future efforts to build large spatiotemporal datasets for algorithmic correction, an initial feature selection approach was performed prior to evaluating overall model performance. Feature selection was performed in a randomly selected 20% subset of the data to avoid overfitting prior to later model evaluation steps. Within this subset, we evaluated both the mean absolute SHAP values as a measure of global feature importance, within a full model with all 25 candidate features, and a recursive stepwise procedure. We adopted 5-fold cross validation split by MAIAC stations to alleviate overfitting to spatial features of the relatively low number of unique stations. In each round of cross validation, backwards feature selection was applied to rank and remove the features by increasing importance. Starting with the XGBoost model containing all 25 candidate features, the overall RMSE was calculated from the out-of-sample predictions after cross validation. Then the feature importance was ranked by mean absolute SHAP values for all the features in the model from low to high. This step was repeated removing the least important feature at each step. After plotting the overall RMSE from the cross-validated predictions against the number of features, we selected the model with the lowest RMSE for Aqua and Terra separately. We then pooled the set of top-ranked features from both satellites to facilitate comparisons between the Aqua and Terra models examined in the full dataset.

**Figure 2.**
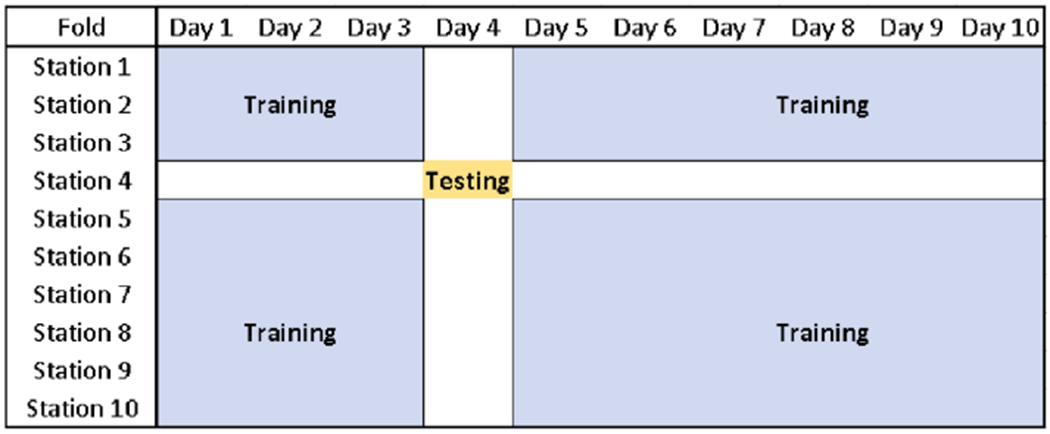
Example of training (blue) and testing (brown) datasets of 1 fold in 10-by-10-fold cross validation. Prediction models are only evaluated on days and at stations that were not used in model training to avoid overfitting.

Using the selected features, grouped 10-by-10-fold cross validation randomly splitting the data by both station and day was performed on the whole dataset. In each training iteration, all observations from 1 fold of stations and from 1 fold of days were withheld with the remaining dataset containing roughly 0.9 × 0.9 = 81% of the training data (a similar share of training data to that in 5-fold cross validation). However, for each combination of withheld data, predictions for evaluating model performance and the corresponding SHAP values were only made at the intersection of withheld days and monitors (~ 1% of the data). Thus predictions for each observation were made on a model trained without any observations from the same day or station (see Fig. 2). For comparison, we also evaluated model performance using grouped 5-fold cross validation separately splitting the data by station or by day. Hyperparameter tuning of the XGBoost model was performed separately in each round of cross validation.

While we used an aggregated measure of the mean absolute SHAP value for each feature as a measure of feature importance in our variable selection, we also plotted the out-of-sample SHAP in order to aid model interpretability. In particular, we plotted frequencies of SHAP values by variable and in bivariate scatterplots versus observed values.

Finally, we conducted an additional external validation of our final model by comparing both the original MAIAC CWV and our corrected CWV with an independent dataset of CWV measured by GPS-based stations in the SuomiNet dataset (Ware et al., 2000), within our Northeastern USA study region – many of which are quite distant from the AERONET sites. All SuomiNet stations use precision survey-quality dual-frequency GPS receivers and antennas. The water-lag-derived CWV measurements from GPS-based stations are generally considered to have excellent precision (5%–10%), exceeding those from sun photometers (Pérez-Ramírez et al., 2014).

## 4 Results

### 4.1 Descriptive analysis of CWV and ΔCWV

**Figure 3.**
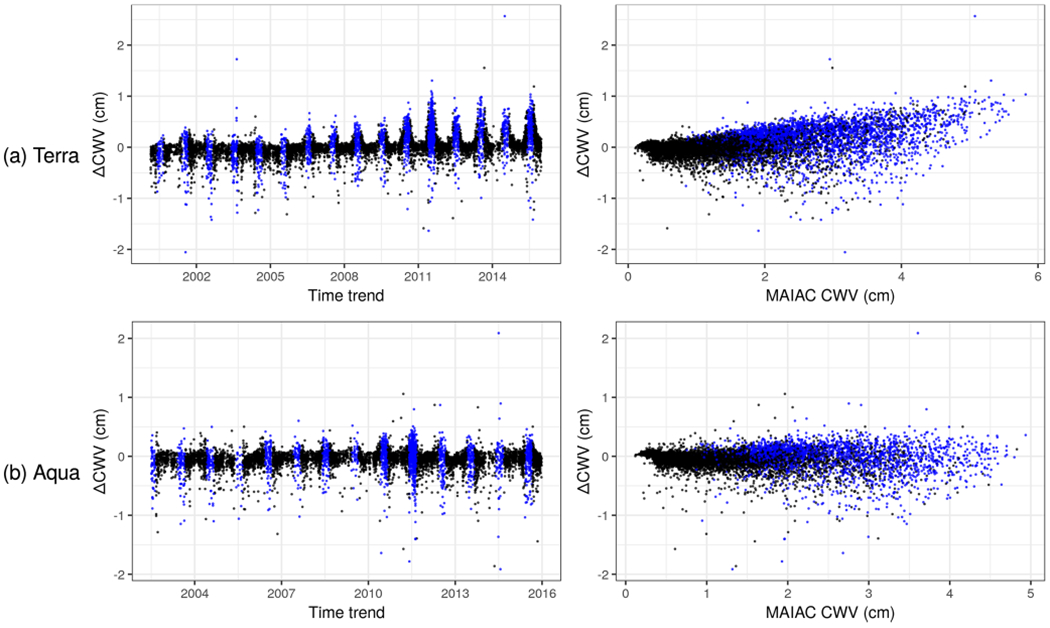
Scatterplots of ΔCWV versus time trend and MAIAC CWV in Terra (**a**) and Aqua (**b**). Observations in the summer months (June–August) are colored in blue.

The overall agreement of the original MAIAC CWV and AERONET CWV was quite good with a Pearson’s correlation of 0.976 and 0.984 for Terra and Aqua, respectively, in agreement with the global MAIAC CWV validation (Martins et al., 2019). However, outlying values and a positive bias in Terra-derived MAIAC CWV particularly indicate a potential for improvement in MAIAC CWV relative to AERONET. The target parameter of ΔCWV (based on the difference between MAIAC and AERONET) was approximately symmetrically distributed and had a mean of 0.043 and −0.054 cm, and a standard deviation of 0.25 and 0.18 cm for the collocated Terra and Aqua datasets, respectively (Table 1). Descriptive scatterplots of the ΔCWV versus individual predictors showed some clear patterns prior to modeling (Figs. 3 and S1 in the Supplement). For example, there is a clear seasonal pattern with a larger SD of ΔCWV in the summer (0.32 and 0.25 cm for Terra and Aqua) when the SD of AERONET CWV is also highest (0.90 cm). This seasonal pattern and the positive bias for Terra (MAIAC CWV overestimates AERONET CWV) is seen to grow larger in more recent years (e.g., 2010–2015). This trend is related to the trend in MODIS Terra calibration, as previously reported (Martins et al., 2017).

### 4.2 Feature selection and model performance

Variable selection using feature importance from SHAP was run in a 20% subset for both Terra and Aqua datasets. Using both global feature importance from a full model and a stepwise backward selection calculating RMSE at each step after ranking variable importance by mean absolute SHAP, we selected six features for the Terra model and selected seven features for the Aqua model. The four features shared by both models were time trend (date represented as an integer), MAIAC CWV, blue band uncertainty, and MAIAC AOD. The other variables selected for Terra were elevation and distance to major water body and for Aqua were the proportion of forest in a 1 km × 1km square, relative azimuth angle, and the proportion of developed area in a 1 km × 1 km square. Pooling these features from both satellites brought the original set of 25 features down to a more parsimonious set of 9 with little loss of model performance (results not shown).

Using the reduced feature set, we implemented the cross validation in the full dataset to evaluate model performance. In the collocated Terra dataset, the predicted ΔCWV evaluated with the grouped monitor-by-day cross validation (10 × 10-fold) explained 45.0% (*R*^2^) of the variance in ΔCWV and reduced the RMSE from 0.252 cm (the root mean squared difference between MAIAC and AERONET CWV) to 0.184 cm, a 26.9% decrease in RMSE. In the collocated Aqua dataset, the predicted ΔCWV explained 24.1% of the variance in ΔCWV (R^2^) and reduced the RMSE from 0.189 to 0.158 cm, a 16.5% decrease in RMSE.

The evaluation of model performance was substantively different depending on how the cross-validation strategy reflected the data structure. Ignoring the nonindependence of the training data by site and withholding unique days for grouped 5-fold cross validation (training on 80% of the data), RMSE for Terra was 0.146 and for Aqua was 0.145 (Table 2), a much better performance (smaller RMSE) that indicates overfitting to the particular sites in the training dataset. Similarly, the RMSE from cross validation split by station (and not by day) was also slightly lower than the RMSE from station-by-day cross validation, suggesting a much smaller degree of overfitting also to the specific dates in the training set.

**Figure 4.**
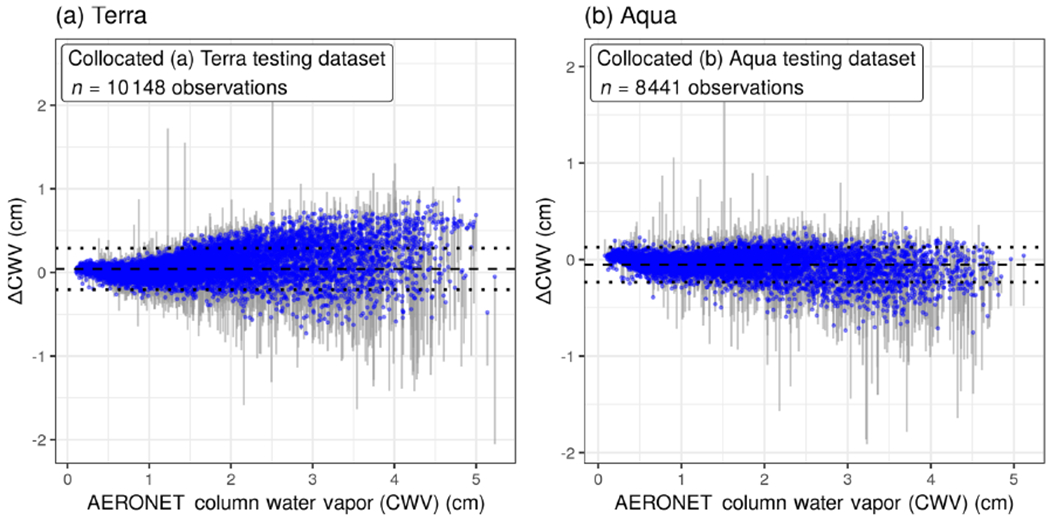
The difference between MAIAC and AERONET CWV values (ΔCWV) was reduced in cross validation of collocated (**a**) Terra and (**b**) Aqua data. The corrected values of ΔCWV are shown with blue points; segments connect back to the measurement error from the raw ΔCWV. The dotted lines show 1 standard deviation from the mean (the dashed line near zero).

After applying the XGBoost model, the measurement error of ΔCWV was corrected to be closer to zero, particularly for the largest-magnitude ΔCWV values. For Terra and Aqua, respectively, 87% and 93% of the ΔCWV observations beyond 1 standard deviation (outside of the dotted lines in Fig. 4, making up 24% of the collocated observations in Terra and 19% in Aqua) had lower measurement error (|ΔCWV|) by an average magnitude of 41% smaller in Terra and 53% smaller in Aqua after XGBoost correction.

We describe the variation in hyperparameters from XG-Boost models across the 100 runs of the site-by-day 10 × 10-fold cross validation. Greater variation in the selected hyperparameter values across folds with very similar training datasets may indicate a lower impact on model performance (Table S2).

### 4.3 Variable importance assessment

Although the final model had already been restricted to include only the top variables from our variable selection approach, we further interpreted variable importance and the contribution of these variables with SHAP values estimated in the grouped cross validation (at monitors and on days not included in the training data for each fold). SHAP values describe the additive contribution to the prediction from every variable for each observation.

**Figure 5.**
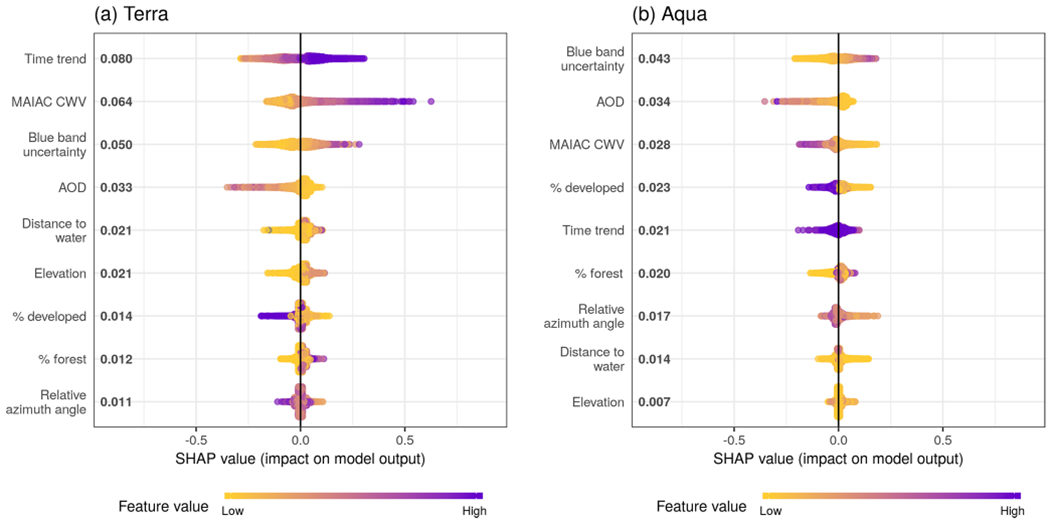
Sina plots show the distribution of feature contributions to predictions of CWV measurement error using SHAP values of each feature for every observation. The *x* axis is set between −1 and 1 to facilitate comparison across subpanels showing models for Terra and Aqua datasets. Features were ordered on the y axis by their mean absolute SHAP values over all observations (bold on the right of the variable names, units are the same as ΔCWV predictions in centimeters). The color is scaled to the feature value (purple high, yellow low).

The SHAP overview plot illustrated different patterns of feature importance in Terra and Aqua (Fig. 5). The rank of the mean absolute SHAP values suggested that the top key contributing variables to predicting the magnitude of ΔCWV in the Terra dataset were time trend (even though all of the data in the testing set were from days not included in the training data, there was still clear seasonality when plotting the SHAP estimates), the magnitude of the MAIAC CWV itself, the blue band uncertainty estimate from MAIAC, the MAIAC AOD, the distance to the nearest major water body, and the elevation. For the Aqua dataset, the blue band uncertainty ranked at the top, followed by the MAIAC AOD, the MAIAC CWV, the proportion of developed area in a 1 km × 1km square, time trend, the proportion of forest coverage in a 1 km × 1 km square, and the relative azimuth angle. The SHAP values ranged from −0.52 to 0.82 cm for Aqua and from −0.55 to 0.30 cm for Terra, aligning with the higher overall error in the Terra dataset.

**Figure 6.**
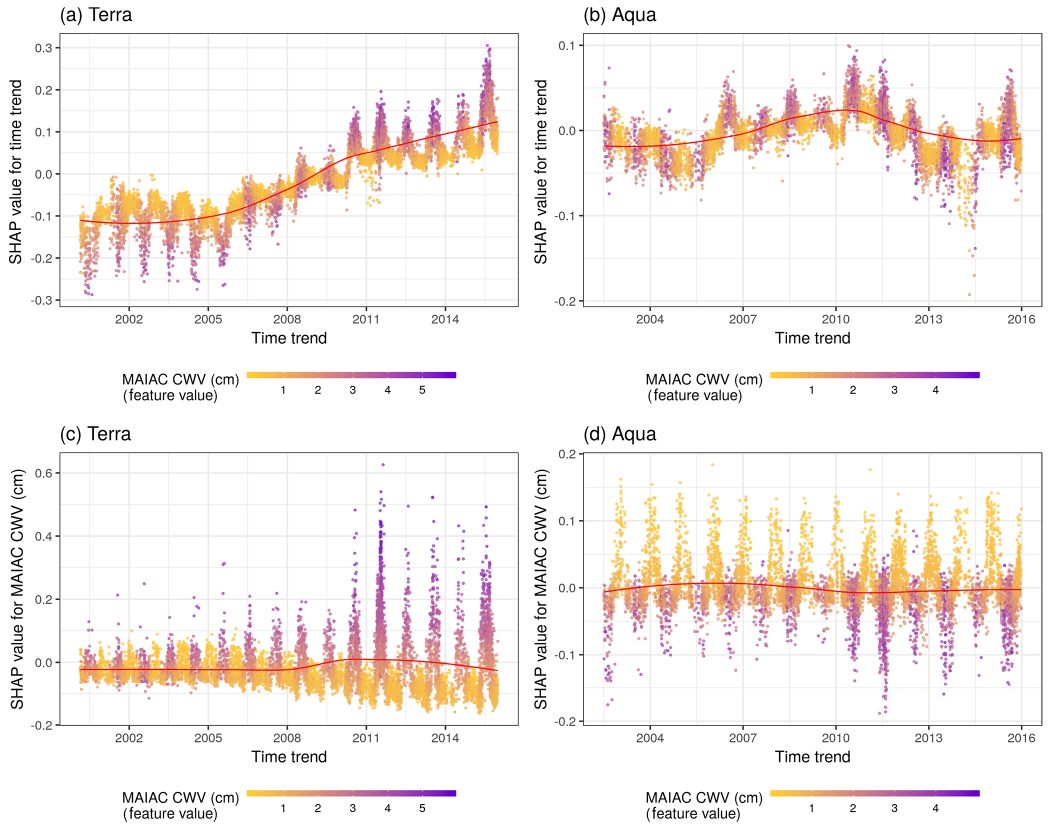
SHAP values showing the contribution of the time trend to predictions for Terra (**a**) and Aqua (**b**). The color represents the MAIAC CWV for each observation (purple high, yellow low). The LOESS (locally estimated scatterplot smoothing) curve is overlaid in red. Terra (**c**) and Aqua (**d**) SHAP values showing the contribution of the MAIAC CWV to predictions of CWV measurement error shown across the time period of the study. Note distinct *y*-axis scales for Terra and Aqua datasets. The color represents the MAIAC CWV for each observation (purple high, yellow low).

For Terra, predicted ΔCWV values became larger in more recent years (Fig. 6a). This suggests the observed positive bias has been getting stronger since ~ 2010. This trend was not observed in Aqua, for which the time trend was a much weaker predictor. Similarly, a higher MAIAC CWV was also more likely to generate higher ΔCWV in Terra (a positive bias), and the influence was becoming stronger along the time trend (Fig. 6b). In contrast, in Aqua the model suggested that MAIAC CWV conservatively underestimated extreme values in both seasons, although the overall impact was weaker (SHAP values closer to zero) and more stationary across time compared to Terra.

**Figure 7.**
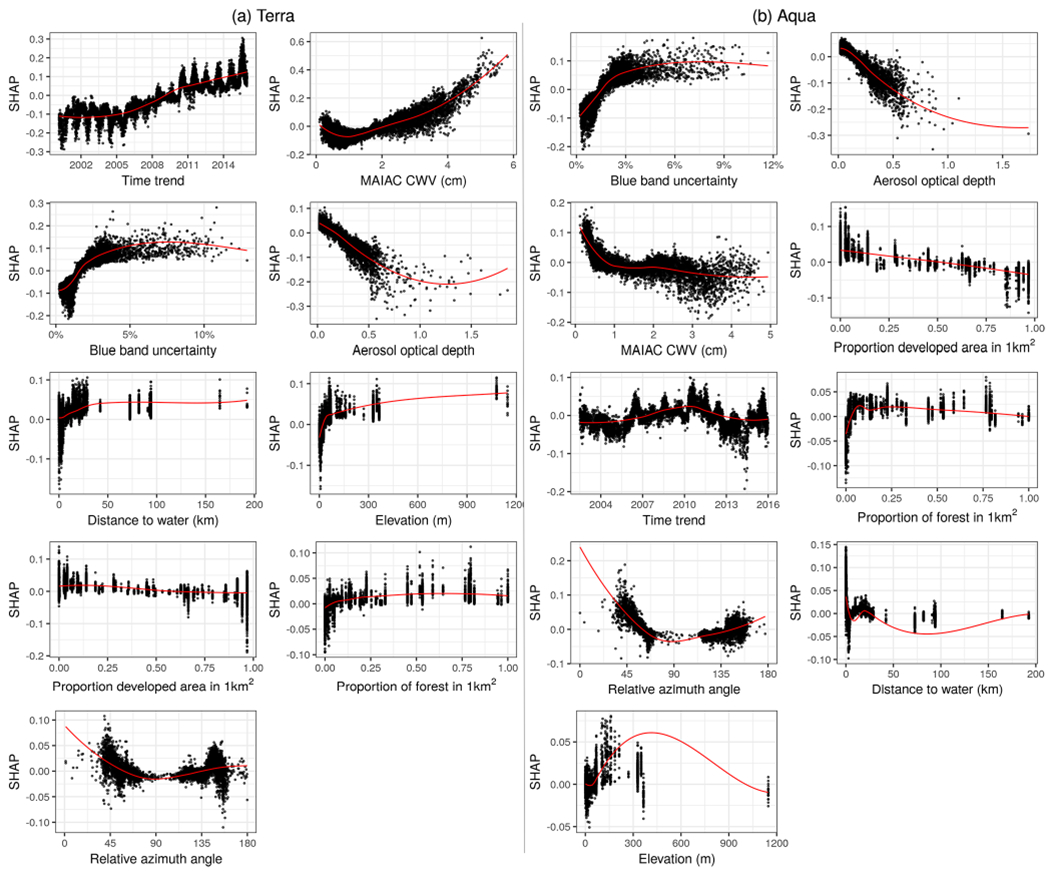
Descriptive scatterplots of the features versus their SHAP scores approximating their contribution to the predictions for ΔCWV (cm) on the *y* axis. Subplots are ordered by overall variable importance (mean absolute SHAP score; see Fig. 5) by satellite.

The impact of the rest of the features was similar for both Terra and Aqua (Fig. 7). Some outlying large AOD values had negative effects on the ΔCWV. Larger blue band uncertainty, higher elevation, or a relative azimuth angle around 45 and 145° increased the error. The SHAP estimates of global feature importance and individual datum contributions clearly diagnose two main factors: (1) changing calibration of MODIS Terra NIR bands at 940 nm over time, resulting in a trend of CWV bias from Terra, and (2) growing underestimation of CWV with increase in AOD. MAIAC CWV retrieval neglects the effect of aerosol scattering, which increases the measured radiances and the band ratios, resulting in underestimation of CWV.

### 4.4 Prediction with new data

To predict into a new dataset, we refit our XGBoost models by again running our nested random hyperparameter tuning using DART tree dropout, this time on the entire training dataset. For both models fit to the Aqua and Terra datasets, the optimal set of hyperparameters (selected from the same set of 50 candidates) was the same, including both L1 and L2 regularization (alpha and lambda), the deepest trees we permitted (maximum depth of 9), and no more dropout (rate drop of 0) than the minimal random selection of one tree per model that had been fixed a priori (with the one drop option; Table 3).

The resulting trained algorithm can generate ~ 3 million MAIAC CWV measurement error estimates per minute (on four cores) in new locations using the XGBoost predict function, and these can be subtracted from the MAIAC CWV value to generate a corrected CWV estimate for downstream use.

### 4.5 Validation with SuomiNet GPS CWV

As an external validation, we applied our XGBoost models to MAIAC data in 1 km × 1 km grid cells containing SuomiNet GPS stations (COSMIC Program Office, 2018). We removed about 20 observations (0.1% of the merged datasets) with outlying CWV values above 9 cm which were almost all from SuomiNet site P776 in year 2011. The resulting validation dataset with collocated Terra or Aqua MAIAC CWV and SuomiNet CWV included 17 469 and 16 466 observations, respectively, from 57 SuomiNet stations (from years 2005 to 2015). SuomiNet CWV in the Terra collocated dataset had a mean of 1.57 ± 1.04 cm, while the Aqua collocated dataset had a mean of 1.50 ± 1.01 cm. The SuomiNet CWV had a more right-skewed distribution than MAIAC CWV.

**Figure 8.**
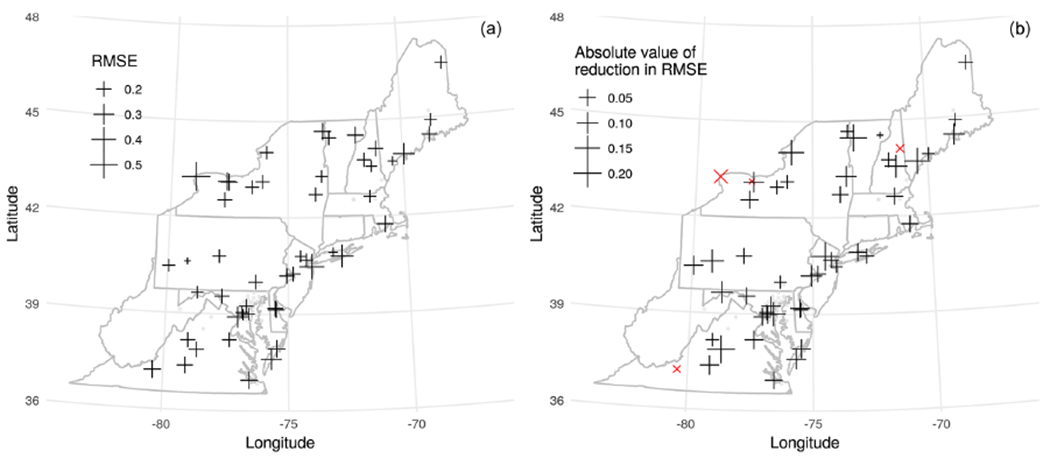
(**a**) RMSE between algorithmically corrected Terra MAIAC CWV and GPS-based CWV for each SuomiNet station shown as crosses. AERONET sites used to train the model are shown as points. (**b**) The difference in RMSE versus GPS CWV using the corrected MAIAC CWV relative to using the original MAIAC CWV by SuomiNet station. The four sites (out of 57 total) which have a higher (worse) RMSE after correction are shown with red × symbols.

After applying our correction, the MAIAC CWV had a lower RMSE versus SuomiNet CWV compared with the raw MAIAC CWV in 53 out of 57 sites for Terra and 56 out of 57 sites for Aqua. The RMSE for agreement with SuomiNet CWV in the full validation dataset improved by 19.7% from 0.28 to 0.22 cm for Terra and by 9.5% from 0.25 to 0.23 cm for Aqua. The Pearson’s correlations of MAIAC CWV with SuomiNet CWV were improved by 1 percentage point from 0.969 to 0.978 for the Terra collocated dataset and by 0.4 percentage points from 0.974 to 0.978 for the Aqua dataset. Plotting the RMSE after correction at SuomiNet locations (grid cells where we make predictions), we observed higher RMSE (worse performance) near Lake Ontario and on the Atlantic coastline (Fig. 8a). Most sites show improved RMSE after correction except four sites for Terra and one site for Aqua (Fig. 8b).

Another goal of addressing measurement error in satellite retrievals is to improve the comparability of different instruments. Given changing atmospheric conditions within the same day between overpass times (Terra in late morning and Aqua in early afternoon), we use the corresponding two SuomiNet CWV measurements to estimate the expected agreement. When restricting to days with both Terra and Aqua CWV observations collocated with SuomiNet observations, we had 9940 station days with all four measurements. Raw MAIAC CWV had a Pearson correlation of 0.975 between Terra and Aqua, and after applying our correction this increased to 0.977, although this was still slightly below that of the two corresponding within-day SuomiNet CWV measurements with a correlation of 0.982. We demonstrate that our algorithmic correction slightly improves on the already excellent agreement of MAIAC CWV from Terra versus Aqua, but is still not quite as close as comparing pairs of within-day measurements from the same ground instruments.

## 5 Discussion

The Northeastern USA exhibits large seasonal variation in CWV. While satellite retrievals using the MAIAC algorithm are overall excellent at estimating CWV, they also have seasonality in their measurement error versus ground measurements from AERONET sun photometers. We show this measurement error has notable heteroscedasticity (larger errors with greater CWV) and has been worsening, with time, for data derived from Terra. Satellite retrievals using MODIS and similar platforms have considerable strengths for measurement of CWV based on their global daily coverage and reconstruction of longer-term records during the satellite era. Our analysis demonstrates that gradient boosting with XG-Boost and features including satellite retrieval quality assurance, aerosol optical depth estimates, land use terms, and time trends can substantially refine satellite-derived retrievals of 1 km × 1 km resolution CWV compared with sun photometer measurements of CWV on test days and at sites that were withheld from training data. Even with this rigorous cross validation, our model explains 45.0% of the measurement error in Terra CWV (*R*^2^) and 24.1% of the measurement error in Aqua CWV. This is an impressive proportion of the difference in MAIAC and AERONET CWV to explain given that the MAIAC CWV is already quite accurate with a ΔCWV standard deviation of only 0.252 and 0.189 cm for Terra and Aqua, respectively, in spite of comparing a 1 km × 1 km satellite retrieval with point measurements from the AERONET sun photometers.

Strategies for model training and cross validation of powerful algorithmic predictive methods need to reflect the structure of the underlying data and the intended use of prediction models – otherwise overfitting may lead to an inaccurate assessment of model performance. Given the sparsity of the collocated AERONET data, we decided to assess performance in cross validation that mimicked the desire to predict in new places (without AERONET stations) and on dates without AERONET data (e.g., when sun photometers are out of service for recalibration).

While our XGBoost models are complex ensembles of 100 boosted regression trees, we use the powerful new SHAP method for interpretation of the importance of each variable and their contributions to individual predictions. Contextualizing the magnitude of the SHAP value (for each variable) and examining the SHAP-based contribution in visualizations along with the feature value distribution can also hint at where retrieval algorithms can be modified for better results. For example, although the measurement error was lower for Aqua, scatterplots for the top two variables by SHAP suggest that MAIAC may underestimate CWV when the blue band uncertainty is very low and may underestimate CWV at higher AOD values. For Terra, the date as an integer is the most important feature, even though our cross-validation approach meant that all SHAP values were estimated for predictions made on dates that did not occur in the training data. Based on the SHAP plots, the date predictor describes seasonal and long-term trends related to an emerging positive bias for Terra that is worse in the summertime.

Demonstrating that there is an improvement in the agreement of corrected MAIAC CWV with the SuomiNet measurements is a strong validation for several reasons. First, the SuomiNet stations offer a well-validated measurement of CWV that relies on a different principle (tropospheric delay) from the sun photometry of the AERONET and the MODIS satellite retrieval of the MAIAC algorithm. The second strength of this validation is that the SuomiNet validation occurred at locations that are unique (not included in the training data from AERONET sites), including many that are far away from the largely coastal AERONET stations in the Northeastern USA. Although Terra CWV also had a larger measurement error versus SuomiNet CWV measurements than Aqua CWV, after our correction using XGBoost, the updated MAIAC CWV for Terra and Aqua both had lower RMSE values of 0.221 and 0.226 cm versus SuomiNet stations – suggesting that we may have achieved parity and perhaps reached the limits of this approach to correct for the sources of measurement error we considered in comparing this satellite retrieval product with point measurements from ground stations.

Strengths of our empirical machine-learning approach include a fast algorithm that uses only a few variables, primarily those already included in the MAIAC retrieval suite and derived land use terms, to correct measurement error in CWV. Limitations of using MODIS-derived CWV from MAIAC include the availability of few measurements per day (versus geostationary satellites) and restriction to cloud-free and daytime values. Our measurement error model has not yet been evaluated for how well it would have worked in a region with substantially fewer AERONET stations or very different climate conditions.

## 6 Conclusions

Empirically correcting for measurement error with machine-learning algorithms is a relatively easy postprocessing opportunity to improve satellite-derived CWV data quality for Earth science and remote sensing applications. Furthermore, the use of machine-learning interpretation tools points to potential sources of measurement error (e.g., a positive bias in CWV retrievals from Terra that has become worse in more recent years) that can help when refining satellite retrieval strategies. We demonstrate that a parsimonious nine-predictor XGBoost model for updating satellite-based column water vapor from the MAIAC retrieval based on AERONET values can decrease measurement error as validated at an independent network of ground sensors across the Northeastern USA.

## Supplementary Material

Supplementary Material

## Figures and Tables

**Table 1.** Descriptive statistics of MAIAC, AERONET CWV, and the ΔCWV for Terra and Aqua by season.

Terra mean±SD (cm)	Spring (*N* = 2265)	Summer (*N* = 3150)	Fall (*N* = 3101)	Winter (*N* = 1632)	Total (*N* = 10148)
MAIAC CWV	1.18±0.82	2.71±0.94	1.39±0.82	0.56±0.30	1.62±1.12
AERONET CWV	1.20±0.80	2.54±0.90	1.40±0.79	0.60±0.34	1.58±1.04
ΔCWV	−0.014±0.192	0.172±0.322	−0.009±0.202	−0.032±0.095	0.043±0.249
Aqua mean±SD (cm)	Spring (*N* = 1921)	Summer (*N* = 2276)	Fall (*N* = 2715)	Winter (*N* = 1529)	Total (*N* = 8441)

MAIAC CWV	1.12±0.74	2.49±0.84	1.33±0.73	0.55±0.27	1.45±0.98
AERONET CWV	1.16±0.78	2.52±0.90	1.42±0.78	0.60±0.33	1.51±1.01
ΔCWV	−0.049±0.150	−0.027±0.253	−0.086±0.159	−0.047±0.101	−0.054±0.181

Note that means and standard deviations (units of centimeters) are shown for 3-month seasons (spring: MAM; summer: JJA; fall: SON; winter: DJF) across all the years and the total.

**Table 2.** Predictive performance in the testing dataset comparing three cross-validation strategies.

	Terra dataset	Aqua dataset
Overall variation	SD 0.25 cm	SD 0.19 cm
Split by day (5-fold)	RMSE 0.15 (57.8%), *R*^2^ = 65.6%	RMSE 0.14 (76.3%), *R*^2^ = 36.5%
Split by station (5-fold)	RMSE 0.18 (71.4%), *R*^2^ = 47.5%	RMSE 0.14 (76.3%), *R*^2^ = 36.5%
Split by station and day (10 × 10-fold)	RMSE 0.18 (73.1%), *R*^2^ = 45.0%	RMSE 0.16 (83.5%), *R*^2^ = 24.1%

Note that the relative percentage of the RMSE compared to overall variation (SD) is listed beside the RMSE.

**Table 3.** Hyperparameters for the Fully Trained XGBoost Model.

Selected values	
eta	0.44
max_depth	9
gamma	0.099
lambda	38
alpha	0.0023
rate_drop	0
one_drop (fixed a priori)	True
nrounds (fixed a priori)	100
