# Supplementary material for: Gradient boosting machine learning to improve satellite-derived column water vapor measurement error
Source: Atmos Meas Tech. Author manuscript; Available in PMC 2020 Nov 13. (PMC7665162; doi:10.5194/amt-13-4669-2020)
Supplement: Supplementary Material [file NIHMS1642057-supplement-Supplementary_Material.pdf]

Supplement of Atmos. Meas. Tech., 13, 4669–4681, 2020
https://doi.org/10.5194/amt-13-4669-2020-supplement
© Author(s) 2020. This work is distributed under
the Creative Commons Attribution 4.0 License.

Atmospheric Measurement Techniques
Open Access

Supplement of

Gradient boosting machine learning to improve satellite-derived column water vapor measurement error

Allan C. Just et al.

Correspondence to: Allan C. Just (allan.just@mssm.edu)

The copyright of individual parts of the supplement might differ from the CC BY 4.0 License.

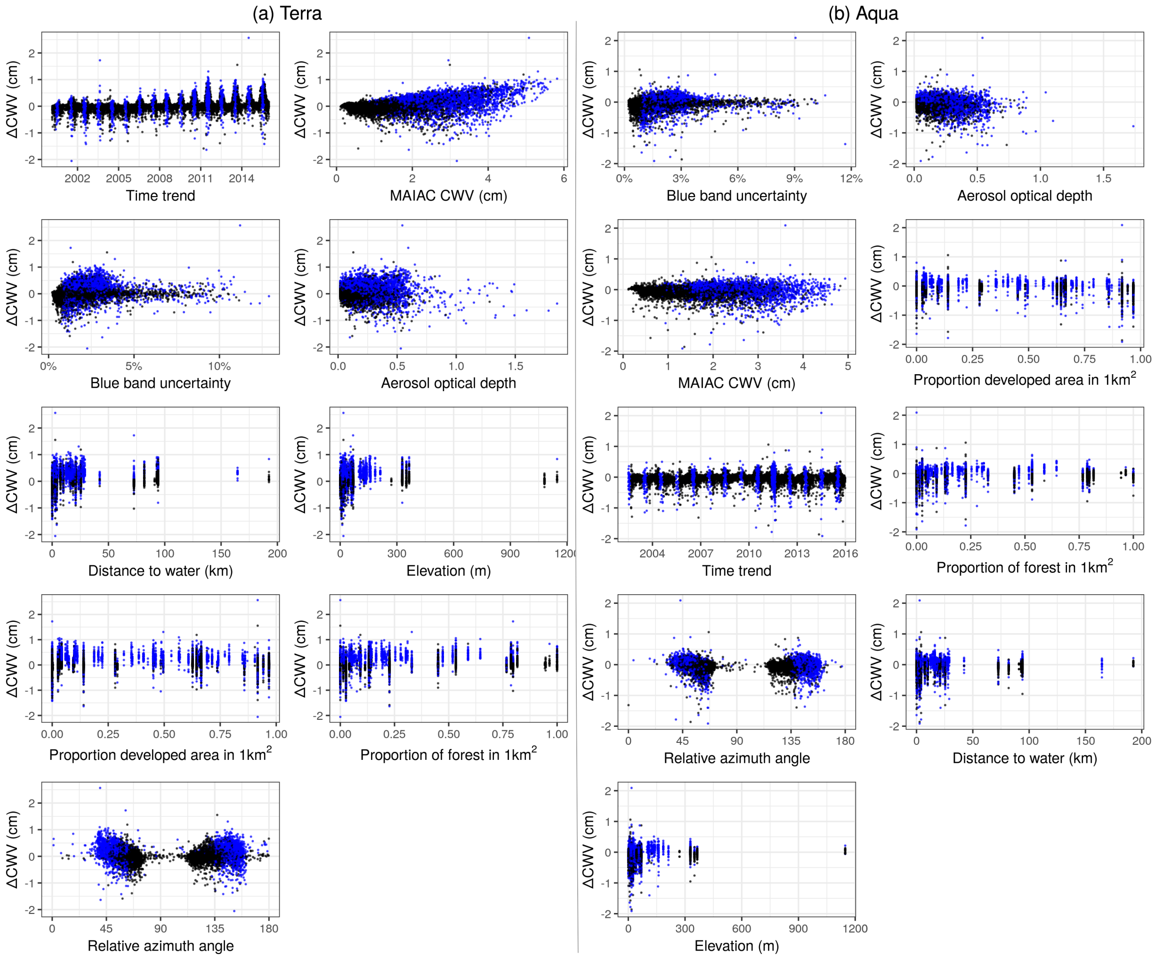

Figure S1. Bivariate scatterplots for all features from the final model versus the difference between MAIAC and AERONET CWV (in cm). Observations from summer months (June-August) are colored in blue.

Table S1. Covariate information for all predictors considered prior to feature selection

Predictor	Data Source	Processing
CWV	MAIAC	Restricted to cells with non-missing AOD (clear sky conditions)
AOD uncertainty (Blue band uncertainty)	MAIAC	
AOD	MAIAC	
Relative azimuth angle	MAIAC	
Elevation	National Elevation Dataset	Aggregated to mean within 1km * 1km grid
Distance to major water body	National Land Cover Dataset (NLCD) 2011	
Proportion of forest	National Land Cover Dataset (NLCD) 2011	Proportion within 1km * 1km grid
Proportion of developed area (all developed categories)	National Land Cover Dataset (NLCD) 2011	Proportion within 1km * 1km grid
Time trend		Integer date
Proportion of water within 5km buffer	National Land Cover Dataset (NLCD) 2011	
Proportion of water within 10km buffer	National Land Cover Dataset (NLCD) 2011	
Proportion of water within 15km buffer	National Land Cover Dataset (NLCD) 2011	
Area of contiguous non-missing MAIAC AOD	MAIAC	R clump function to detect adjacent non-missing raster cells
Number of non-missing observations in focal window 3km * 3km	MAIAC	R focal function with square window
Number of non-missing observations in focal window 30km * 30km	MAIAC	R focal function with square window
Number of non-missing observations in focal window 50km * 50km	MAIAC	R focal function with square window
Number of non-missing observations in focal window 110km * 110km	MAIAC	R focal function with square window
Number of non-missing observations in focal window 210km * 210km	MAIAC	R focal function with square window

(Continued) **Predictor**	**Data Source**	**Processing**
Number of non-missing observations in focal window 310km * 310km	MAIAC	R focal function with square window
Number of non-missing observations in focal window 410km * 410km	MAIAC	R focal function with square window
Number of non-missing observations in focal window 510km * 510km	MAIAC	R focal function with square window
Proportion of low developed area	National Land Cover Dataset (NLCD) 2011	Proportion within 1km * 1km grid
Proportion of medium developed area	National Land Cover Dataset (NLCD) 2011	Proportion within 1km * 1km grid
Proportion of high developed area	National Land Cover Dataset (NLCD) 2011	Proportion within 1km * 1km grid
Proportion of open space developed area	National Land Cover Dataset (NLCD) 2011	Proportion within 1km * 1km grid

Note. **The full model included 25 predictors prior to feature selection. Predictors were generated from MAIAC auxiliary and quality control fields, time trend, elevation, and land use. The top 9 predictors were selected into the models.**

Table S2. XGBoost hyperparameters selected in the 100 rounds of grouped ten-by-ten-fold cross-validation

Terra	**mean**	**median**	**sd**	**min**	**max**
eta	0.406	0.44	0.065	0.23	0.46
max_depth	8.4	9	1.206	6	9
gamma	0.076	0.099	0.034	0.017	0.099
lambda	26.142	38	17.008	0.004	38
alpha	0.074	0.002	0.168	0.002	0.56
rate_drop	0.003	0	0.006	0	0.025

Aqua	**mean**	**median**	**sd**	**min**	**max**
eta	0.405	0.44	0.081	0.11	0.46
max_depth	8.79	9	0.769	6	9
gamma	0.087	0.099	0.024	0.017	0.099
lambda	29.813	38	15.509	0.004	38
alpha	0.102	0.002	0.241	0.002	1.2
rate_drop	0.001	0	0.005	0	0.025

Note. **The parameter nrounds was set to 100 and one_drop was set to true a priori.**
